# Vertebral body dosimetry associated with hematologic toxicities and survival in esophageal squamous-cell carcinoma treated with chemoradiotherapy plus immunotherapy

**DOI:** 10.3389/fimmu.2026.1832306

**Published:** 2026-07-31

**Authors:** Shijie Shang, Qian Song, Xinyi Liang, Shan Yin, Zijun Zhai, Shuling Ma, Xinpei Li, Haonan Xiao, Jingxin Zhang, Jinming Yu, Jiachun Ma, Xiaorong Dong, Dawei Chen

**Affiliations:** 1 Cancer Center, Union Hospital, Tongji Medical College, Huazhong University of Science and Technology, Wuhan, China; 2 Institute of Radiation Oncology, Union Hospital, Tongji Medical College, Huazhong University of Science and Technology, Wuhan, China; 3 Hubei Key Laboratory of Precision Radiation Oncology, Wuhan, China; 4 Shandong Provincial Key Laboratory of Precision Oncology, Shandong Cancer Hospital and Institute, Shandong First Medical University and Shandong Academy of Medical Sciences, Jinan, China; 5 Cheeloo College of Medicine, Shandong University, Jinan, China; 6 Department of Radiation Oncology and Physics, Shandong Cancer Hospital and Institute, Shandong First Medical University and Shandong Academy of Medical Sciences, Jinan, China

**Keywords:** esophageal squamous-cell carcinoma, radiotherapy, vertebral body dosimetry, lymphopenia, immunotherapy, survival

## Abstract

**Background:**

The addition of immunotherapy to definitive chemoradiotherapy has improved outcomes for patients with unresectable advanced esophageal squamous-cell carcinoma (ESCC). However, treatment-related hematologic toxicity, particularly radiation-induced lymphopenia, may impair systemic antitumor immunity and compromise the efficacy of immunotherapy. Because vertebral bodies (VB) contain a substantial proportion of active bone marrow, irradiation of this region may contribute to immune cell depletion. This study evaluated the association between VB dose–volume parameters and grade ≥3 hematologic toxicities (HT3+) in ESCC patients treated with chemoradiotherapy and immunotherapy.

**Methods:**

All radiation doses were converted to equivalent doses in 2 Gy fractions (EQD_2_). Vertebral bodies were contoured, and VB dose–volume parameters (V5_EQD2_–V50_EQD2_, Dmean_EQD2_, and Dmax_EQD2_) were extracted. Logistic regression was used to evaluate factors associated with HT3 +. Receiver operating characteristic (ROC) analysis was performed to determine optimal VB dose thresholds. These thresholds were subsequently evaluated for associations with post-radiotherapy absolute lymphocyte count (ALC), immunotherapy response, progression-free survival (PFS), and overall survival (OS). Survival outcomes were analyzed using the Kaplan–Meier method.

**Results:**

Among 157 ESCC patients, 141 (89.8%) developed HT3 +. Low-dose VB irradiation parameters (V5_EQD2_–V20_EQD2_) were significantly associated with HT3+, predominantly driven by lymphocyte depletion. ROC analysis demonstrated moderate discriminative ability, with AUCs of 0.704, 0.709, and 0.699 for V5_EQD2_, V10_EQD2_, and V20_EQD2_, respectively. The corresponding optimal thresholds of V5_EQD2_ = 60.1%, V10_EQD2_ = 53.9%, and V20_EQD2_ = 51.9%, which effectively stratified HT3+ risk. Patients meeting these VB dose constraints exhibited higher post-radiotherapy ALC, improved immunotherapy response, and favorable survival outcomes, particularly OS.

**Conclusions:**

Low-dose VB irradiation is strongly associated with severe hematologic toxicity in ESCC patients receiving chemoradiotherapy and immunotherapy. Minimizing VB exposure during treatment planning may help preserve lymphocyte counts, mitigate radiation-induced immune suppression, and support immune-mediated treatment efficacy.

## 1 Introduction

Esophageal cancer (EC) remains a leading cause of cancer-related mortality worldwide (1), with the majority of patients presenting with locally advanced disease at diagnosis. For those with unresectable, locally advanced esophageal squamous-cell carcinoma (ESCC), concurrent chemoradiotherapy (CCRT) remains the standard treatment. Recent phase III trials have demonstrated that the addition of immune checkpoint inhibitors targeting the programmed cell death protein-1 or ligand-1 (PD-(L)1) axis can further improve outcomes in advanced ESCC (2, 3), prompting increasing integration of immunotherapy with definitive CCRT in clinical practice. Early clinical evidence from small cohorts suggests that the integration of immunotherapy with chemoradiotherapy is both feasible and efficacious (4). Radiotherapy and chemotherapy may potentiate immunotherapy through immunogenic cell death and modulation of the tumor microenvironment; however, this intensified multimodality approach also heightens concern regarding treatment-related toxicities that may compromise immune function.

Radiotherapy-induced bone marrow suppression, particularly treatment-related lymphopenia, remains a major clinical challenge. Severe hematologic toxicities can lead to infection complications and transfusion requirements, impairing quality of life and increasing healthcare burden. Moreover, unplanned treatment interruptions or delays due to hematologic toxicities may compromise local tumor control and overall therapeutic outcomes. Previous studies have highlighted that the effective dose of radiation to immune cells is a key factor influencing lymphopenia and survival outcomes in EC following chemoradiotherapy (5, 6). A clinical research analysis revealed that radiotherapy carries a risk of grade ≥3 toxicities, including hematologic adverse events (7). A multicenter randomized trial further demonstrated that neoadjuvant CRT compared to neoadjuvant chemotherapy would increase the incidence of grade ≥3 hematologic toxicities (HT3+) (8). A meta-analysis also identified lymphopenia as the most common HT3+ in patients receiving combined radiotherapy and immunotherapy (9). In the definitive CCRT setting for locally advanced ESCC, radiation exposure to normal tissues has been shown to correlate with profound lymphocyte depletion and inferior clinical outcomes (10). In the current era of immunotherapy, treatment-related hematologic toxicities may have broader implications by modulating systemic immunity and being associated with responsiveness to immune checkpoint inhibitors and survival outcomes. These findings highlight the critical need to identify radiotherapy-related determinants of hematologic toxicities and immune preservation in ESCC patients undergoing CCRT in combination with immunotherapy.

Given the radiosensitivity of bone marrow stem cells, advanced radiotherapy techniques such as intensity-modulated radiotherapy and proton beam therapy have been clinically adopted to mitigate radiation-induced myelotoxicity. A re-analysis of a prospective study demonstrated that reducing irradiation to lymphocyte-related organs, including the spleen and bone marrow, significantly decreases the incidence of severe lymphocyte depletion in ESCC (11). Building on this concept, several studies have investigated dosimetric predictors of hematologic toxicities in ESCC, identifying vertebral body (VB) marrow dose–volume parameters as potential contributors to myelosuppression during chemoradiotherapy (12, 13). However, the relevance of VB dosimetry has not been evaluated in the setting of combined chemoradiotherapy and immunotherapy. Moreover, despite its substantial marrow content, the vertebral column is not routinely delineated as an organ at risk in standard radiotherapy planning. Therefore, this study aimed to characterize the association between VB dose–volume parameters and HT3+ in patients with ESCC treated with chemoradiotherapy and immunotherapy, with the aim of informing marrow-sparing treatment strategies in the era of immunotherapy.

## 2 Materials and methods

### 2.1 Patient cohort and treatment evaluation

This retrospective study enrolled 157 patients with histologically confirmed ESCC who received CCRT combined with immunotherapy at our institution between July 2021 and May 2025. All patients underwent conventional fractionated radiotherapy and received at least two cycles of PD-(L)1 inhibitor therapy. Tumor location was determined through endoscopic evaluation, with all tumors confined to either the cervical or thoracic esophageal segment. Clinical staging was assigned according to the 8th edition of the American Joint Committee on Cancer (AJCC) TNM classification for ESCC, based on integrated imaging assessments. Treatment response to immunotherapy was assessed using the immune-modified Response Evaluation Criteria in Solid Tumors (imRECIST).

### 2.2 Hematologic assessment and toxicity definition

Patient eligibility required baseline hematologic parameters above the threshold for Grade 1 cytopenia, as defined by the *Common Terminology Criteria for Adverse Events* (CTCAE), version 5.0. Serial hematologic parameters, including white blood cell (WBC) count, absolute neutrophil count (ANC), platelet count, hemoglobin level, and absolute lymphocyte count (ALC), were recorded from the initiation of radiotherapy to three months following treatment completion. Acute HT3+ was defined per CTCAE v5.0 criteria as a decline to any of the following thresholds within the observation period: WBC< 2.0 × 10^9^/L, ANC< 1.0 × 10^9^/L, platelet count< 50 × 10^9^/L, hemoglobin< 80 g/L, or ALC< 0.5 × 10^9^/L. Hematologic supportive care was administered only when clinically indicated, and HT grading was based on nadir hematologic values recorded during the observation period.

### 2.3 VB contouring and calculation of EQD_2_ and BED

For all patients, the vertebral bodies were contoured from the C6 to T11 levels. Given the heterogeneity in prescribed dose fractionation schemes, all radiation doses were converted to the equivalent doses in 2 Gy fractions (EQD2) using the linear-quadratic model, where D denotes the total physical dose, d is the dose per fraction, and the α/β ratio was set to 10 Gy. From the resulting dose-volume histograms (DVHs), the following volumetric parameters were extracted: V5_EQD2_, V10_EQD2_, V20_EQD2_, V30_EQD2_, V40_EQD2_, and V50_EQD2_, representing the percentage of VB volumes receiving at least 5, 10, 20, 30, 40, and 50 Gy in EQD2, respectively. The mean and maximum EQD2 doses to the VB were also calculated and denoted as Dmean_EQD2_ and Dmax_EQD2_.


EQD2=D∗(d+α/β)/(2.0+α/β)

To provide a complementary biological assessment, all parameters were additionally computed as biologically effective doses (BED) using the formula, with α/β again set to 10 Gy. This yielded the corresponding BED-based indices (V5_BED_–V50_BED_, Dmean_BED_, and Dmax_BED_) for comparative dosimetric analysis.


BED=D∗[1+d/(α/β)]

### 2.4 Statistical analysis

The primary endpoint was the incidence of acute HT3 +. Secondary endpoints included progression-free survival (PFS) and overall survival (OS). PFS was defined as the interval from treatment initiation to the first documented disease progression or death from any cause, and OS was defined as the interval from treatment initiation to death from any cause. Factors associated with HT3+ and immunotherapy response were assessed using univariate and multivariate logistic regression analyses. Variables with *P* < 0.10 in the univariate analyses were included in the multivariate analyses. Dosimetric parameters were compared between patients with and without HT3+ using the Mann–Whitney U test. Additional comparisons of dosimetric parameters according to tumor length and N stage were performed using the Student’s t-test or Mann–Whitney U test. Spearman correlation analyses were performed to evaluate the relationships between planning target volume (PTV) length and VB dosimetric parameters, as well as between ALC and VB dosimetric parameters. Optimal cut-off values for dosimetric parameters were identified via receiver operating characteristic (ROC) curve analysis, using the maximum Youden’s index. The corresponding area under the curve (AUC) and odds ratio (OR) with 95% confidence intervals (CI) were reported for each parameter. Patients were subsequently stratified according to these cut-off values to evaluate their clinical significance. Post-radiotherapy ALC values were compared with the Mann–Whitney U test, and immunotherapy response rates were analysed with the chi-square test, with complete and partial responses (CR/PR) classified as responders (R) and stable or progressive disease (SD/PD) as non-responders (NR). Survival outcomes were estimated using the Kaplan–Meier method and compared by the log-rank test. Multivariate Cox proportional hazards regression analyses were performed to evaluate the associations between VB dosimetric parameters and survival outcomes. To account for multiple testing in analyses evaluating associations between VB dose–volume parameters and hematologic toxicities, *P* values were adjusted using the Benjamini–Hochberg false discovery rate (FDR) procedure, and FDR-adjusted *P* values (Adj. *P*)< 0.05 were considered statistically significant for these analyses. Because of the strong correlations among VB dose–volume parameters and the substantial overlap between PTV length and VB dose–volume parameters, separate multivariate logistic and Cox proportional hazards regression models were constructed for PTV length, V5_EQD2_, V10_EQD2_, and V20_EQD2_, with each parameter entered individually together with other tumor-related and treatment-related factors. For consistency of presentation, the adjusted estimates of other covariates were presented from the V20_EQD2_ model. Similar results were obtained when V5_EQD2_ or V10_EQD2_ were used as alternative VB dose–volume parameters. All statistical analyses were two-sided, with *P<* 0.05 considered statistically significant. Analyses were conducted using SPSS Statistics version 26.0 (IBM Corp., Armonk, NY, USA), GraphPad Prism version 10.0 (GraphPad Software, Boston, MA, USA) and R version 4.2.1 (R Foundation for Statistical Computing, Vienna, Austria).

## 3 Results

### 3.1 Patient characteristics

Baseline characteristics of the 157 enrolled patients are summarized in **Table 1**. The cohort was predominantly male (n = 139, 88.5%). Of the 157 patients, 79 (50.3%) had a tumor length ≤5 cm and 78 (49.7%) had a tumor length >5 cm. Regarding nodal burden, 67 patients (42.7%) were classified as AJCC N0–1 and 90 patients (57.3%) as N2 **–** 3. Most primary tumors were located in the thoracic esophagus (n = 125, 79.6%). Anti–PD-1 immune checkpoint inhibitors were administered in 140 patients (89.2%), while 17 patients (10.8%) received anti–PD-L1 inhibitors. The most commonly used chemotherapy regimen was nab-paclitaxel combined with platinum-based agents (n = 124, 79.0%).

**Table 1:** Baseline characteristics of patients with ESCC.

Variables	Value*
**Age, y**	62 (57–68)
Gender	
Male	139 (88.5%)
Female	18 (11.5%)
**BMI(kg/m^2^)**	22.0 (20.0–25.4)
ECOG	
0	78 (49.7%)
1	79 (50.3%)
Smoking history	
No	62 (39.5%)
Yes	95 (60.5%)
Drinking history	
No	66 (42.0%)
Yes	91 (58.0%)
Tumor length	
≤5cm	79 (50.3%)
>5cm	78 (49.7%)
T stage	
T1	3 (1.9%)
T2	15 (9.6%)
T3	119 (75.8%)
T4	20 (12.7%)
N stage	
N0-1	67 (42.7%)
N2-3	90 (57.3%)
Tumor site	
Cervical	17 (10.8%)
Thoracic	125 (79.6%)
Both	15 (9.6%)
Baseline blood counts	
WBC (10^3^/μL)	6.0 (5.2–7.5)
ANC (10^3^/μL)	3.7 (2.8–4.8)
Platelets (10^3^/μL)	212 (173–257)
Hemoglobin (g/μL)	13.0 (12.2–14.2)
ALC (10^3^/μL)	1.6 (1.4–2.0)
Immunotherapy type	
Anti-PD-1 immune checkpoint	140 (89.2%)
Anti-PD-L1 immune checkpoint	17 (10.8%)
Immunotherapy timing	
Before, during, and after RT	48 (30.6%)
Before and after RT	20 (12.7%)
Before RT alone	17 (10.8%)
After RT alone	29 (18.5%)
Concurrent RT	43 (27.4%)
Immunotherapy response	
Response	52 (33.1%)
Non-response	105 (66.9%)
Chemotherapy regimen	
Paclitaxel ± Platinum	17 (10.8%)
Nab-paclitaxel ± Platinum	124 (79.0%)
Docetaxel ± Platinum	7 (4.5%)
Others	9 (5.7%)
**PTV length (cm)**	18.00 (14.10–21.75)
**Prescription dose (converted into EQD_2_, Gy)**	53.1 (49.6–58.4)
TVB DVH (converted into EQD_2_)	
V5_EQD2_ (%)	62.1 (48.4–85.9)
V10_EQD2_ (%)	59.6 (46.2–78.8)
V20_EQD2_ (%)	55.2 (42.2–72.1)
V30_EQD2_ (%)	40.9 (29.8–51.5)
V40_EQD2_ (%)	19.4 (5.9–33.5)
V50_EQD2_ (%)	0.1 (0.0–13.1)
Dmean_EQD2_ (Gy)	22.5 (16.8–26.2)
Dmax_EQD2_ (Gy)	48.6 (43.2–54.7)

ESCC, esophageal squamous-cell carcinoma; BMI, body mass index; ECOG, Eastern Cooperative Oncology Group; WBC, white blood cells; ANC, absolute neutrophil count; ALC, absolute lymphocyte count; PD-1, programmed cell death protein 1; PD-L1, programmed cell death ligand 1; RT, radiation therapy; PTV, planning target volume; EQD_2_, equivalent total dose in 2-Gy fraction; TVB, thoracic vertebral body; DVH, dose-volume histogram.

*Values are number (percentage) or median (IQR).

Median VB dose–volume parameters were V5_EQD2_ 62.1%, V10_EQD2_ 59.6%, V20_EQD2_ 55.2%, V30_EQD2_ 40.9%, V40_EQD2_ 19.4%, and V50_EQD2_ 0.1%. The median Dmax_EQD2_ and Dmean_EQD2_ were 48.6 Gy (interquartile range (IQR), 43.2 **–** 54.7) and 22.5 Gy (IQR, 16.8 **–** 26.2), respectively.

### 3.2 Factors and DVH parameters associated with HT3+

Among the 157 patients, 141 (89.8%) developed HT3+ during or after radiotherapy. The incidences of HT3+ for leukopenia, neutropenia, thrombocytopenia, anemia, and lymphopenia were 39.5%, 36.9%, 5.1%, 1.9%, and 83.4%, respectively (**Supplementary Table 1**). Univariate and multivariate logistic regression analyses were performed to identify clinical factors associated with HT3 +. As summarized in **Table 2**, PTV length was significantly associated with HT3+ in the univariate analysis and remained an independent predictor in the multivariate analysis. No significant associations were observed between HT3+ and baseline characteristics, including age, gender, body mass index (BMI), Eastern Cooperative Oncology Group (ECOG) performance status, smoking or drinking history, tumor length, N stage, baseline hematologic parameters, treatment regimens (immunotherapy and chemotherapy), or prescribed radiation dose. An extended analysis incorporating additional clinical characteristics similarly revealed no significant correlations with HT3+ (**Supplementary Table 2**).

**Table 2:** Univariate and multivariate analyses of factors associated with HT3+.

Variables	Univariate analysis		Multivariate analysis	
	OR (95% CI)	*P* value	OR (95% CI)	*P* value
Age	0.980 (0.920–1.045)	0.546		
Gender				
Male vs Female	1.116 (0.232–5.366)	0.891		
BMI	1.045 (0.902–1.211)	0.558		
ECOG				
1 vs 0	0.767 (0.271–2.713)	0.617		
Smoking history				
Yes vs No	0.670 (0.221–2.031)	0.479		
Drinking history				
Yes vs No	1.081 (0.381–3.067)	0.884		
Tumor length				
>5cm vs ≤5cm	1.739 (0.600–5.043)	0.308		
N stage				
N2–3 vs N0-1	1.050 (0.370–2.978)	0.927		
Baseline WBC	1.038 (0.818–1.318)	0.758		
Baseline ANC	1.076 (0.805–1.440)	0.620		
Baseline Platelets	1.001 (0.994–1.009)	0.754		
Baseline Hemoglobin	0.853 (0.604–1.204)	0.366		
Baseline ALC	0.666 (0.244–1.812)	0.426		
Immunotherapy type				
Anti-PD-L1 vs anti-PD-1	0.833 (0.172–4.027)	0.821		
Immunotherapy timing				
Before, during, and after RT	Reference		Reference	
Before and after RT	0.391 (0.051–2.992)	0.366	0.707 (0.085–5.870)	0.748
Before or after RT	0.357 (0.066–1.938)	0.232	0.544 (0.095–3.118)	0.494
Concurrent RT	0.224 (0.044–1.142)	0.072	0.399 (0.073–2.197)	0.291
Chemotherapy regimen				
Nab-paclitaxel + Platinum	Reference			
Paclitaxel + Platinum	0.409 (0.100–1.668)	0.213		
Docetaxel + Platinum	0.526 (0.058–4.815)	0.570		
Others	0.307 (0.056–1.679)	0.173		
PTV length	1.240 (1.080–1.424)	0.002	1.221 (1.060–1.406)	0.006^†^
Prescription dose (converted into EQD_2_)	1.043 (0.986–1.013)	0.146		

BMI, body mass index; ECOG, Eastern Cooperative Oncology Group; WBC, white blood cells; ANC, absolute neutrophil count; ALC, absolute lymphocyte count; PD-1, programmed cell death protein 1; PD-L1, programmed cell death ligand 1; RT, radiation therapy; PTV, planning target volume; EQD_2_, equivalent total dose in 2-Gy fraction.

^†^*P* value is significant.

**Figure 1:**
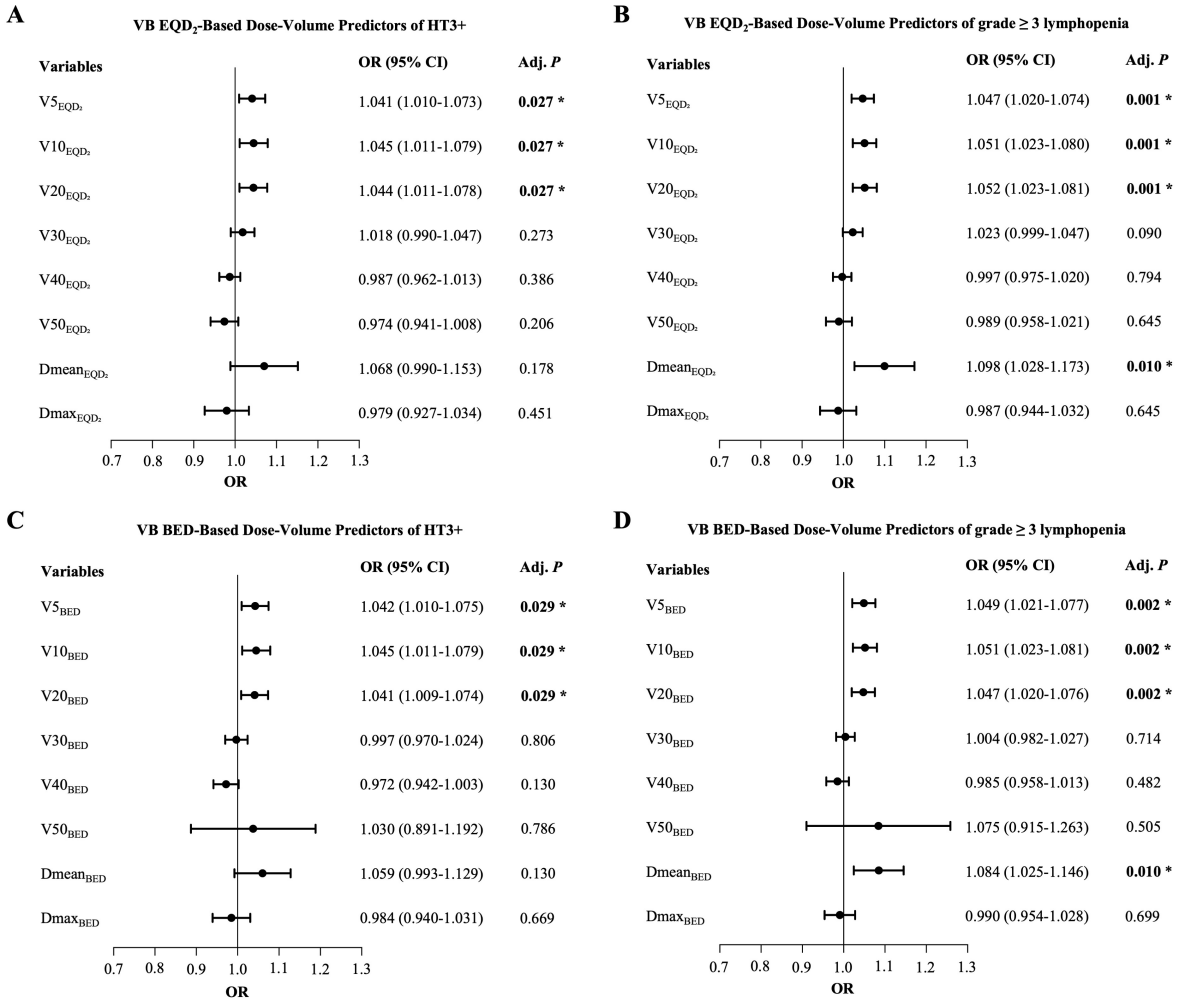
Forest plots of VB dose–volume parameters associated with HT3+ and lymphopenia. Forest plots illustrate the associations between VB EQD2-based dose–volume parameters and **(A)** HT3+ and **(B)** grade ≥3 lymphopenia. **(C, D)** Corresponding analyses based on BED-converted parameters demonstrate consistent relationships. Each parameter is presented with its risk ratio and 95% confidence interval. OR, odds ratio; VB, vertebral bone; HT3+, grade ≥3 hematologic toxicity; EQD2, equivalent dose in 2 Gy fraction; BED, biologically effective dose.

**Figure 2:**
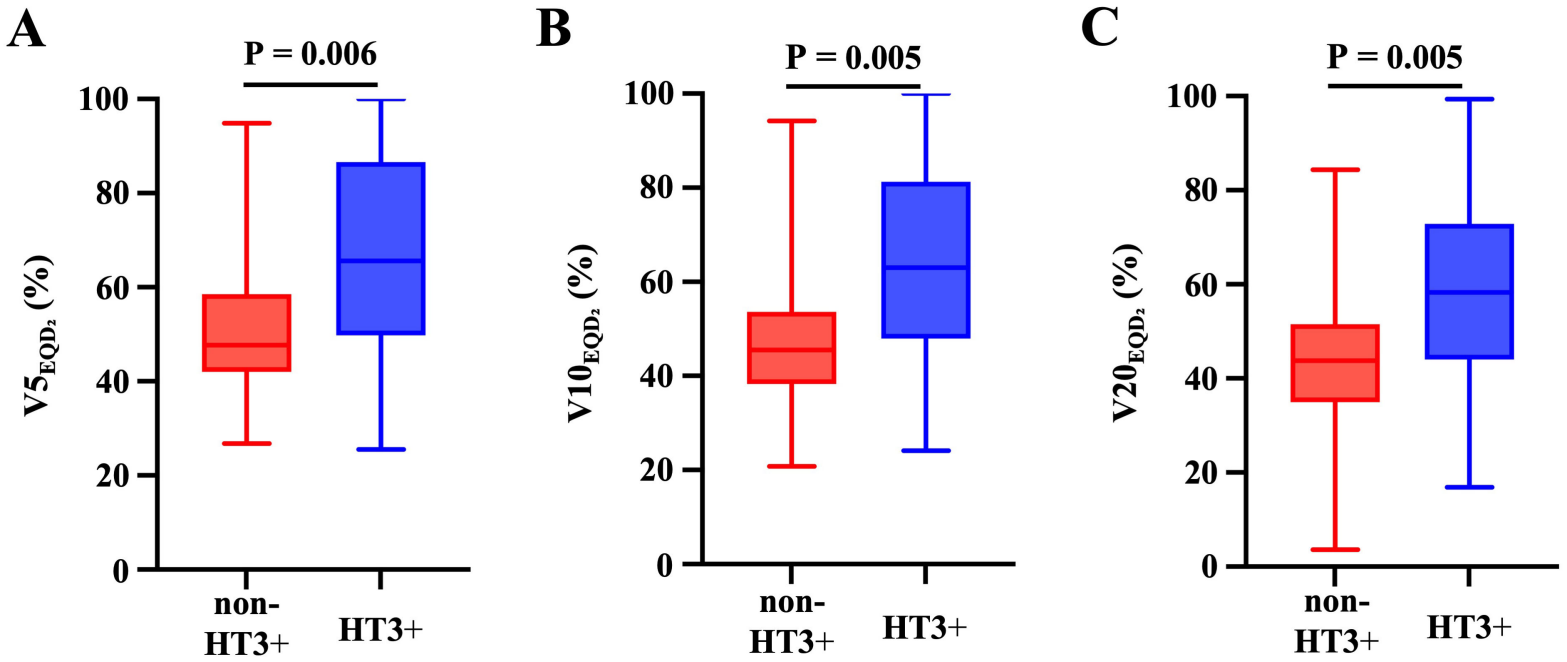
VB dose–volume parameters according to HT3+ status. Box plots depict VB **(A)** V5_EQD2_, **(B)** V10_EQD2_, and **(C)** V20_EQD2_ values in patients who developed HT3+ compared with those who did not. VB, vertebral bone; HT3+, grade ≥3 hematologic toxicity; EQD_2_, equivalent dose in 2 Gy fraction.

In contrast, several VB dose–volume parameters were significantly associated with HT3 +. On logistic regression analysis, higher VB V5_EQD2_, V10_EQD2_, and V20_EQD2_ were associated with an increased risk of HT3+ (all Adj. *P<* 0.05; **Figure 1A**). Patients who developed HT3+ had significantly higher V5_EQD2_, V10_EQD2_, and V20_EQD2_ values compared to those who did not (**Figures 2A–C**). To further evaluate the relationship between irradiated target extent and VB dose exposure, additional exploratory analyses were performed. Spearman correlation analyses demonstrated that PTV length was strongly and positively correlated with VB low-dose irradiation parameters, including V5_EQD2_ (r = 0.844, *P* < 0.001), V10_EQD2_ (r = 0.862, *P* < 0.001), and V20_EQD2_ (r = 0.850, *P* < 0.001). In addition, tumor length and N stage were significantly associated with VB low-dose irradiation parameters (V5_EQD2_, V10_EQD2_, and V20_EQD2_) (**Supplementary Table 3**).

Given that grade ≥3 lymphopenia was the predominant contributor to HT3+, sensitivity analyses were performed using grade ≥3 lymphopenia as an alternative endpoint. Consistent with the primary HT3+ analysis, significant associations remained for V5_EQD2_, V10_EQD2_, V20_EQD2_ and Dmean_EQD2_ (all Adj. *P* < 0.05; **Figure 1B**). Additional lineage-specific analyses were performed for other hematologic toxicities. No significant associations were identified between VB dose–volume parameters and leukopenia or neutropenia (**Supplementary Figures 1A, B**). All VB dose–volume parameters were significantly associated with thrombocytopenia (**Supplementary Figure 1C**). Likewise, no significant associations were observed with anemia (**Supplementary Figure 1D**).

Analysis based on BED-converted parameters yielded consistent results. Low-dose VB parameters (V5_BED_–V20_BED_) remained significantly associated with HT3+ (all Adj. *P<* 0.05; **Figure 1C**), while V5_BED_–V20_BED_ and Dmean_BED_ were associated with lymphopenia (all Adj. *P<* 0.05; **Figure 1D**). For lineage-specific hematologic toxicities, no significant associations were identified between BED-converted VB parameters and leukopenia or neutropenia (**Supplementary Figures 1E, F**). Associations between BED-based parameters and thrombocytopenia remained significant (**Supplementary Figure 1G**). In contrast, V40_BED_ and V50_BED_ were significantly associated with anemia (**Supplementary Figure 1H**).

### 3.3 Association of VB dosimetry with HT3+ and immunotherapy response

**Figure 3:**
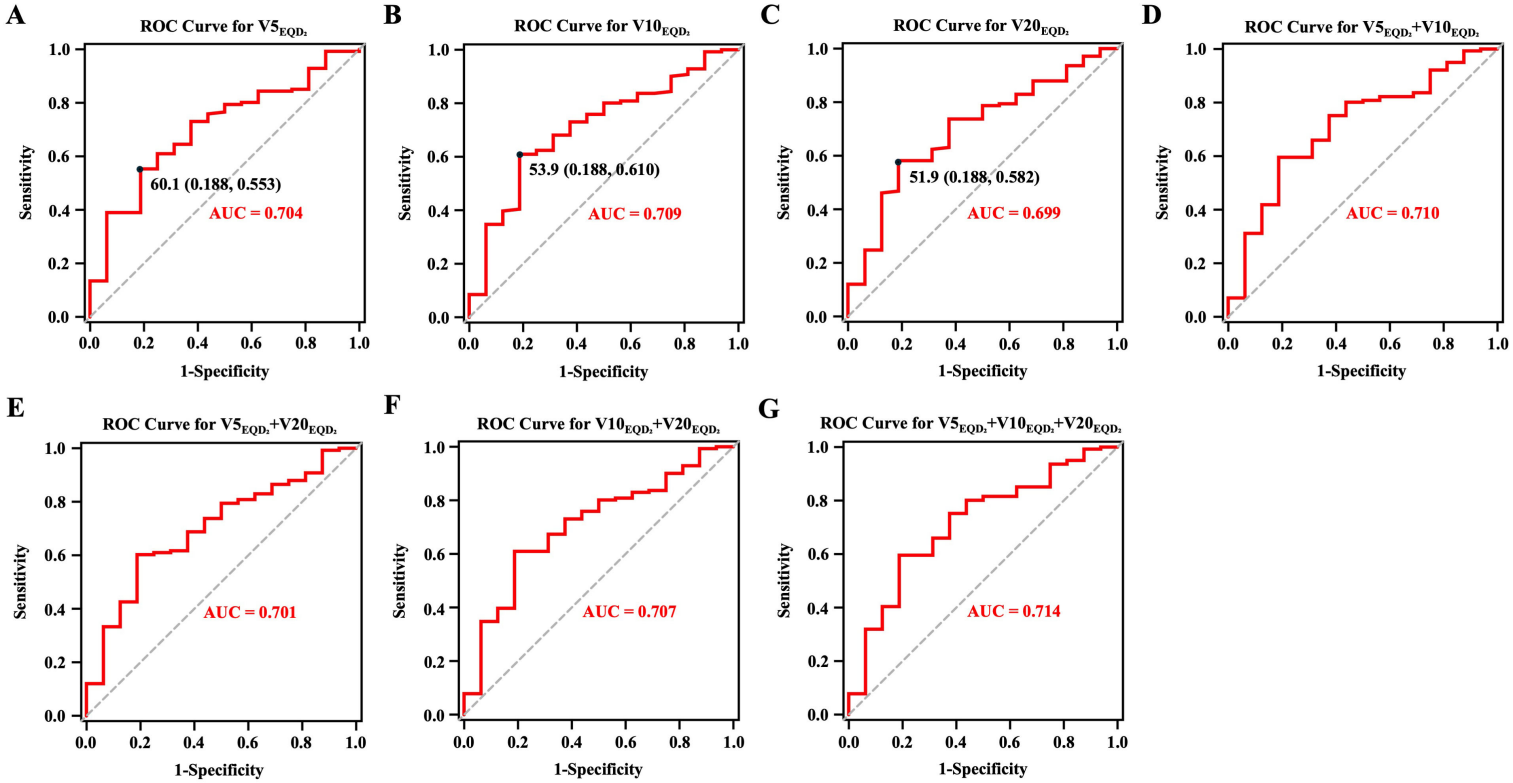
ROC curves for VB dose–volume parameters associated with HT3 +. ROC curves illustrate the discriminative ability of **(A)** V5_EQD2_, **(B)** V10_EQD2_, and **(C)** V20_EQD2_ for identifying HT3 +. Arrows indicate the optimal cutoff points determined by maximum Youden’s index. ROC analyses of composite models for identifying HT3+ are shown in **(D)** V5_EQD2_ + V10_EQD2_, **(E)** V5_EQD2_ + V20_EQD2_, **(F)** V10_EQD2_ + V20_EQD2_, and **(G)** V5_EQD2_ + V10_EQD2_ + V20_EQD2_. VB, vertebral bone; HT3+, grade ≥3 hematologic toxicity; EQD_2_, equivalent dose in 2 Gy fraction.

ROC curve analysis was used to evaluate the discriminative ability of V5_EQD2_, V10_EQD2_, and V20_EQD2_ for HT3 +. The corresponding AUCs were 0.704, 0.709, and 0.699, respectively (**Figures 3A–C**). A composite model incorporating all three parameters demonstrated improved discrimination compared with individual metrics (**Figures 3D–G**). The optimal cut-off values derived from ROC curve analysis were V5_EQD2_ = 60.1%, V10_EQD2_ = 53.9%, and V20_EQD2_ = 51.9% (**Figures 3A–C**). Patients exceeding these thresholds had a significantly higher risk of developing HT3+ with OR of 5.365 (95% CI, 1.464–19.657; *P* = 0.011) for V5_EQD2_, 6.776 (95% CI, 1.846–24.867; *P* = 0.004) for V10_EQD2_, and 6.023 (95% CI, 1.643–22.081; *P* = 0.007) for V20_EQD2_.

**Figure 4:**
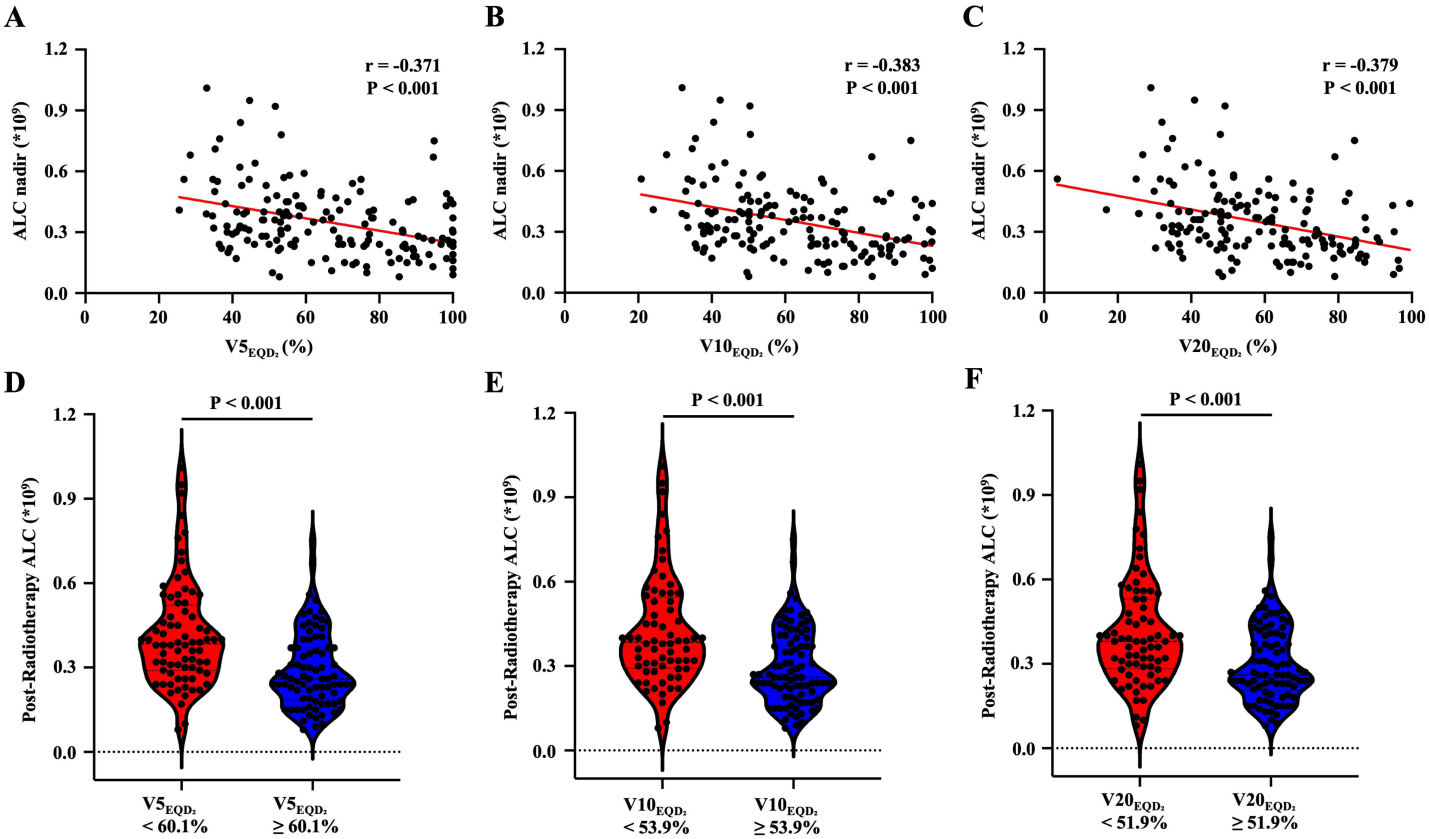
Association between VB dose–volume parameters and post-radiotherapy ALC. Spearman correlation analyses depict the relationships between **(A)** V5_EQD2_, **(B)** V10_EQD2_, **(C)** V20_EQD2_ and the post-radiotherapy ALC. Post-radiotherapy ALC are compared between patient groups stratified by the dosimetric cutoffs for **(D)** V5_EQD2_, **(E)** V10_EQD2_, and **(F)** V20_EQD2_, respectively. VB, vertebral bone; ALC, absolute lymphocyte count; EQD_2_, equivalent dose in 2 Gy fraction.

**Figure 5:**
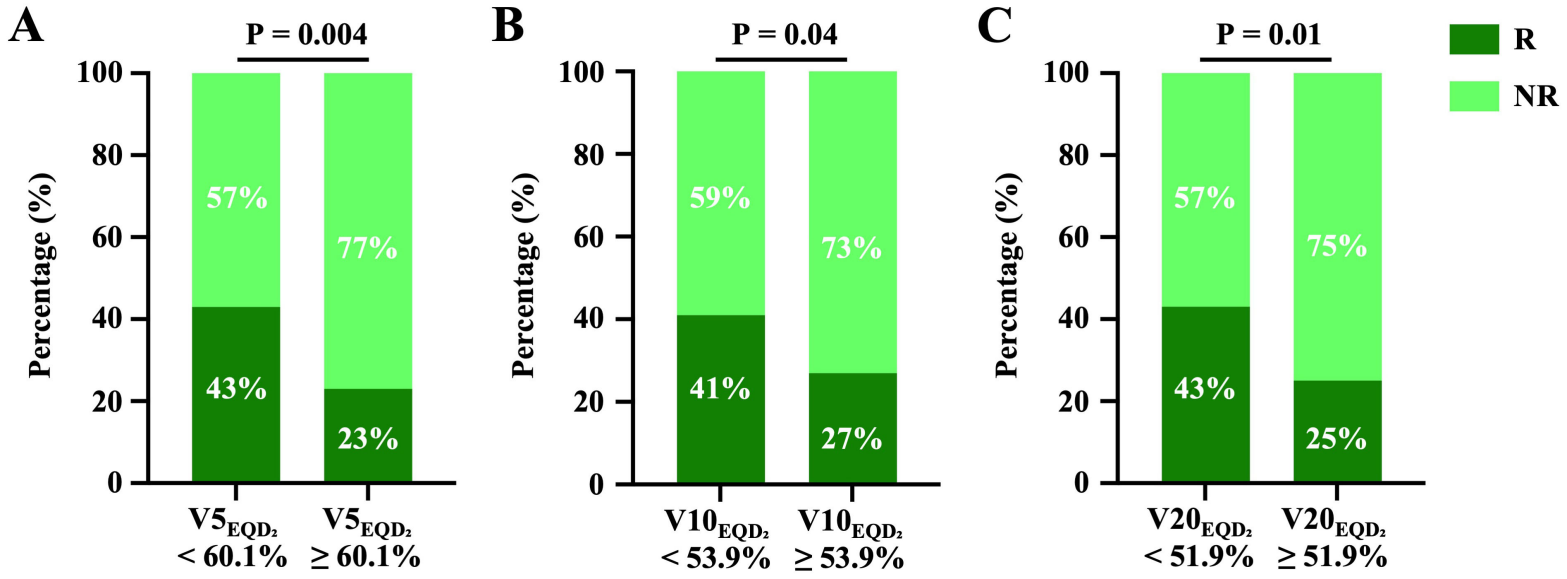
Immunotherapy response according to VB dose constraints. Immunotherapy response rates are shown for the corresponding dose thresholds of **(A)** V5_EQD2_, **(B)** V10_EQD2_, and **(C)** V20_EQD2_. VB, vertebral bone; EQD_2_, equivalent dose in 2 Gy fraction.

Consistent with the findings from the lymphopenia sensitivity analysis, ROC analyses demonstrated comparable discriminative performance for V5_EQD2_, V10_EQD2_, and V20_EQD2_, with AUCs of 0.736, 0.743, and 0.735, respectively (**Supplementary Figure 2**). Spearman correlation analysis revealed significant inverse associations between low-dose VB parameters and post-radiotherapy nadir ALC, including V5_EQD2_ (r = -0.371), V10_EQD2_ (r = -0.383), V20_EQD2_ (r = -0.379) (all *P* < 0.05; **Figures 4A–C**). Consistent findings were observed using BED-converted parameters, with V5_BED_, V10_BED_, and V20_BED_ also showing significant negative correlations with the nadir ALC (**Supplementary Figures 3A–C**). When stratified by the identified dosimetric thresholds, patients with V5_EQD2_< 60.1%, V10_EQD2_< 53.9%, or V20_EQD2_< 51.9% had significantly higher post-radiotherapy ALC levels (all *P* < 0.05; **Figures 4D–F**). Higher immunotherapy response rates were also observed in patients meeting these dose constraints (all *P* < 0.05; **Figures 5A–C**). To further determine whether these associations were independent of clinicopathologic and treatment-related factors, multivariate logistic regression analyses were performed. In the adjusted analyses, sex, stage group, and PTV length were identified as independent factors associated with immunotherapy response. Moreover, all three low-dose VB dosimetric parameters (V5_EQD2_, V10_EQD2_, and V20_EQD2_) remained independently associated with immunotherapy response in the corresponding models (**Supplementary Table 4**).

### 3.4 Survival outcomes according to VB dose thresholds

**Figure 6:**
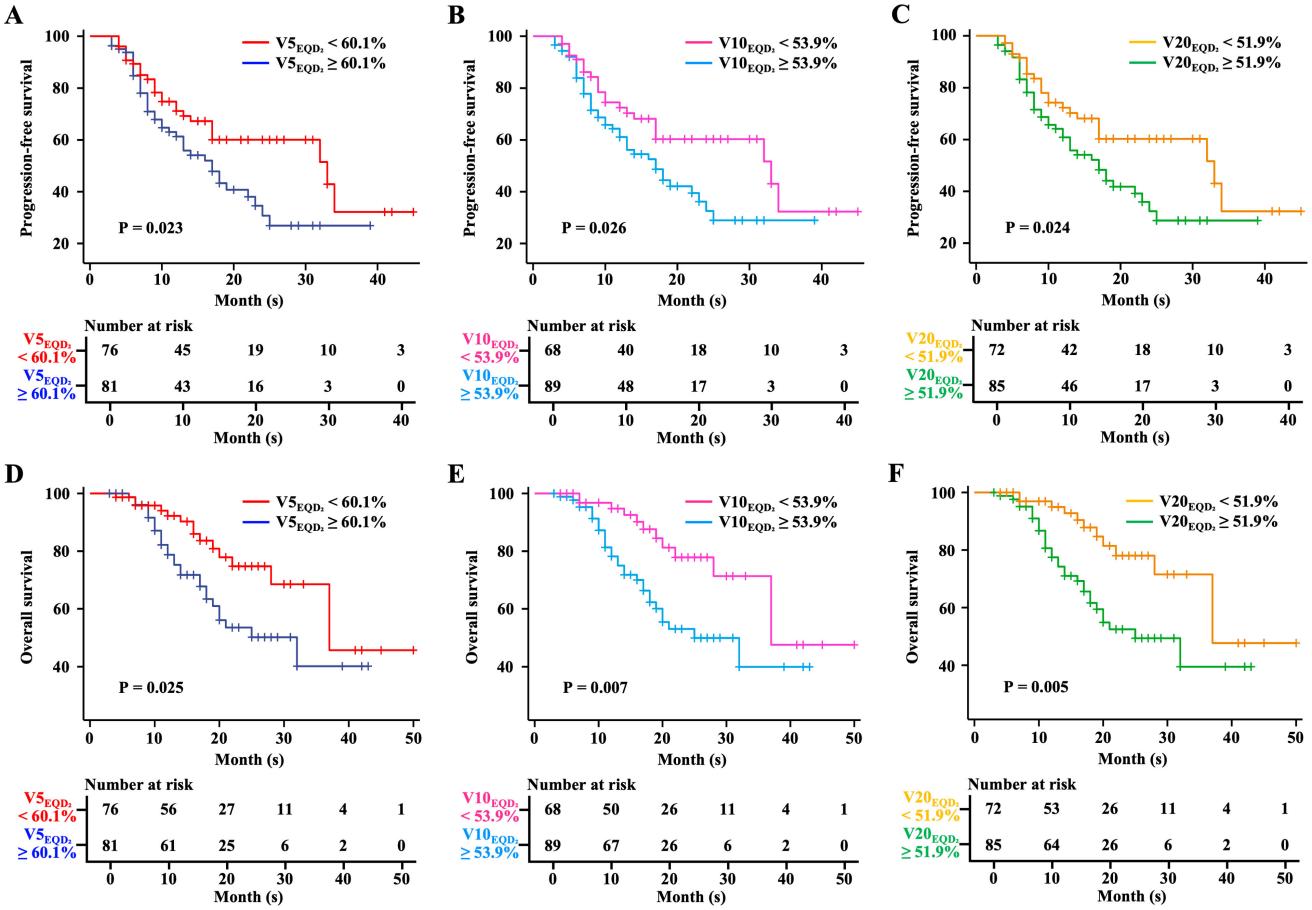
PFS and OS stratified by VB dose–volume thresholds. Kaplan–Meier analyses comparing PFS and OS between patient groups stratified by VB **(A, D)** V5_EQD2_, **(B, E)** V10_EQD2_, and (**C, F**) V20_EQD2_ based on the optimal dosimetric cutoffs. PFS, progression-free survival; OS, overall survival; VB, vertebral bone; EQD_2_, equivalent dose in 2 Gy fraction.

PFS and OS were analyzed according to the identified VB dosimetric thresholds. Patients with VB V5_EQD2_< 60.1% had a significantly longer median PFS compared with those receiving higher VB doses (33.0 vs. 17.0 months; *P* = 0.023; **Figure 6A**). Similar differences in PFS were observed for V10_EQD2_< 53.9% (33.0 vs. 17.0 months; *P* = 0.026; **Figure 6B**) and V20_EQD2_< 51.9% (33.0 vs. 17.0 months; *P* = 0.024; **Figure 6C**). Consistent findings were observed for OS. Patients with VB V5_EQD2_< 60.1% had a longer median OS compared with those with higher VB exposure (37.0 vs. 32.0 months; *P* = 0.025; **Figure 6D**). The median OS was 37.0 months among patients with V10_EQD2_< 53.9%, compared with 25.0 months for those exceeding this threshold (*P* = 0.007; **Figure 6E**). Similarly, patients with V20_EQD2_< 51.9% had a longer OS than those with higher V20_EQD2_ values (37.0 vs. 25.0 months; *P* = 0.005; **Figure 6F**). To further determine whether the observed survival differences were independent of clinicopathologic and treatment-related factors, univariate and multivariate Cox proportional hazards regression analyses were performed. In the adjusted analyses, sex and stage group were independently associated with PFS (**Supplementary Table 5**). For OS, PTV length and low-dose VB dose–volume parameters (V5_EQD2_, V10_EQD2_, and V20_EQD2_) showed independent associations in their respective models (**Supplementary Table 6**).

## 4 Discussion

In this study, we analyzed patients with advanced ESCC treated with CCRT combined with immunotherapy and found that low-dose VB irradiation parameters, particularly V5, V10, and V20, were independently associated with the development of HT3 +. These associations remained significant after adjustment for potential clinical confounders, including age, baseline hematologic parameters, and treatment-related factors, suggesting a consistent association between vertebral marrow irradiation and treatment-related hematologic toxicity in this setting. Notably, VB dose–volume parameters were primarily associated with lymphopenia and thrombocytopenia, whereas no significant associations were observed with leukopenia or neutropenia. This differential pattern suggests that vertebral marrow irradiation was more strongly correlated with lymphoid and megakaryocytic suppression rather than myeloid precursor suppression, potentially reflecting differences in radiosensitivity across hematopoietic compartments. Together, these findings support the relevance of VB dose optimization as a marrow-sparing consideration in patients receiving combined chemoradiotherapy and immunotherapy. The incidence of HT3+ in the present cohort was higher than previously reported in some studies, which was largely driven by grade ≥3 lymphopenia. This may partly be attributable to the inclusion of patients with locally advanced or advanced ESCC receiving concurrent chemoradiotherapy combined with PD-(L)1 blockade, as concurrent chemotherapy and thoracic radiotherapy are known to substantially contribute to lymphocyte depletion and hematologic suppression.

Given the central role of lymphocytes in orchestrating antitumor immune responses, particularly in the context of immune checkpoint blockade, treatment-related lymphopenia holds significant implications beyond merely hematologic toxicity. Previous studies have identified dosimetric predictors of treatment-related lymphopenia in ESCC, primarily within the context of chemoradiotherapy alone (14, 15). Building on this evidence, our findings suggest that low-dose VB irradiation is associated not only with severe HT but also with immune-related treatment outcomes in patients receiving combined chemoradiotherapy and immunotherapy. The observed associations between VB dose exposure, lymphocyte depletion, and immunotherapy response support the biologic plausibility of marrow-sparing radiotherapy strategies in the era of immunotherapy. Bone marrow radiosensitivity has long been established, serving as the foundation for marrow-sparing approaches in conventional chemoradiotherapy for malignancies in the pelvic, abdominal, and thoracic regions (16–20). Our results extend this principle by suggesting that preservation of vertebral marrow could have broader implications, not only in reducing treatment-induced toxicity but also in preserving systemic immune competence, a crucial factor for optimizing responses to immune checkpoint inhibitors.

From a radiobiological perspective, VB marrow behaves is generally considered to behave as a parallel organ, reflecting its composition of multiple redundant functional subunits (21). Consequently, marrow function is influenced more strongly by the cumulative dose delivered to a large volume of marrow rather than by focal high-dose exposure. This volume-dependent dose–response relationship has been consistently reported across various malignancies. In pelvic radiotherapy, for example, the risk of treatment-related myelosuppression has been shown to correlate more closely with low-to-intermediate dose–volume parameters than with maximum dose metrics (16). Similar observations have been reported in non-small cell lung cancer, where the mean VB dose and low-dose irradiation parameters were identified as important correlates of severe hematologic toxicities (22–24). In ESCC, emerging evidence further suggests that proton therapy, by reducing low-dose irradiation of the vertebral column, is associated with a lower incidence of hematologic toxicities (25). Together, these studies provide a radiobiological framework for interpreting our findings, in which HT3+ was more strongly associated with low dose-volume parameters (V5–V20) rather than maximum dose metrics. Given that a substantial proportion of active VB is encompassed within thoracic radiation fields during radiotherapy for ESCC, limiting low-dose irradiation to the vertebral column may represent an important strategy for reducing treatment-related lymphopenia and maintaining systemic antitumor immunity during immunotherapy.

The interaction between anticancer therapy and host immunity is increasingly recognized as a critical determinant of therapeutic efficacy. Among conventional treatment modalities, radiotherapy has been consistently associated with treatment-related lymphopenia across multiple tumor types and disease settings (26–28). In ESCC, radiotherapy-induced lymphopenia has been linked to inferior survival outcomes (29), highlighting the importance of immune preservation during treatment. Given the central role of bone marrow in hematopoiesis and immune cell homeostasis, incidental irradiation of vertebral marrow during thoracic radiotherapy may contribute to HT, particularly in radiosensitive lymphocyte populations. Supporting this concept, prior studies have reported that irradiation of lymphocyte-rich osseous structures, such as the sternum, is associated with significant reductions in peripheral lymphocyte counts in patients with ESCC (30). Similar observations have also been described in non-small cell lung cancer, where higher thoracic vertebral marrow dose was correlated with greater lymphocyte depletion and reduced responsiveness to immunotherapy (23). In addition to vertebral marrow reserve, irradiation of circulating immune cells may also contribute to treatment-related lymphopenia during thoracic radiotherapy. The concept of effective dose to immune cells (EDIC) has been developed to estimate radiation exposure to circulating blood and immune cell populations during treatment. Previous studies have demonstrated that higher EDIC is associated with increased lymphocyte depletion and inferior clinical outcomes in thoracic malignancies (31–33). Within this context, our findings suggest that VB marrow–sparing radiotherapy may help preserve lymphocyte counts during combined chemoradiotherapy and immunotherapy. While causality cannot be inferred, the observed associations between lower vertebral marrow dose, improved immunotherapy response, and favorable survival outcomes support the potential relevance of marrow preservation for maintaining immune competence during multimodality treatment.

Interestingly, the association between VB dose–volume parameters and survival outcomes differed between PFS and OS. After adjustment for tumor-related and treatment-related factors, VB dosimetry remained associated with OS but not PFS. This discrepancy may reflect the distinct biological determinants underlying these endpoints. PFS reflects early disease control and is influenced by multiple clinical factors, including tumor burden, intrinsic tumor characteristics, and treatment-related factors. In our cohort, stage group showed a strong association with PFS, and adjustment for tumor-related factors may have accounted for part of the prognostic information shared between tumor burden and VB dosimetry. In contrast, OS represents a broader clinical endpoint that may additionally capture the long-term effects of treatment-related toxicity, immune function, and subsequent treatment tolerance. Similar observations have been reported in immune checkpoint inhibitor studies, where clinically meaningful OS benefits may occur despite limited improvements in PFS (34–36). Therefore, the persistent association between VB dose–volume parameters and OS may reflect the potential contribution of vertebral irradiation-related hematologic toxicity and immune modulation beyond tumor progression alone.

Several limitations should be considered when interpreting the results of this study. First, the retrospective single-center design and relatively modest sample size may introduce potential selection bias and limit the generalizability of the proposed VB dose thresholds. In addition, VB contouring was performed using a uniform C6–T11 range to improve consistency in vertebral dosimetric assessment rather than being individualized according to individual tumor location or target extent. Although varying marrow-bearing vertebral volumes across different tumor locations may affect the generalizability of the proposed thresholds, the observed associations may still provide a clinically relevant reference. External validation in larger, multi-institutional cohorts is warranted. Second, the relatively modest immunotherapy response rate in our cohort may partly reflect the challenges of radiographic assessment after combined chemoradiotherapy and immunotherapy, where treatment-related inflammation, delayed immune responses, and pseudo-progression may influence imaging interpretation despite independent review according to imRECIST. Third, although induction chemotherapy was not significantly associated with severe HT in univariate analyses, its potential influence on bone marrow reserve cannot be completely excluded. In addition, pathological and molecular biomarker data, including PD-L1 expression and other relevant pathological information, were incompletely available because of the retrospective real-world design and therefore were not incorporated into the multivariable analyses. Future prospective studies incorporating standardized treatment regimens and more comprehensive biomarker data are warranted to further validate the independent association between VB dosimetry and treatment outcomes. Fourth, although vertebral marrow irradiation emerged as a significant dosimetric correlate of HT3+, strategies to reduce VB dose should be balanced against the dose to adjacent organs at risk, including the heart, lungs, and great vessels. Future studies should evaluate VB dose constraints within a comprehensive organ-sparing radiotherapy framework, which may further optimize treatment safety and immune preservation in patients with ESCC. Taken together, the findings of the present study should be interpreted as exploratory and require validation in larger prospective cohorts before being adopted as clinical dose constraints.

## 5 Conclusion

In patients with advanced ESCC receiving CCRT combined with immunotherapy, low-dose irradiation of the VB, particularly V5, V10, and V20, was significantly associated with the development of HT3 +. Specific dose–volume thresholds (V5_EQD2_< 60.1%, V10_EQD2_< 53.9%, and V20_EQD2_< 51.9%) effectively stratified the risk of severe HT and were associated with higher post-radiotherapy lymphocyte counts as well as improved clinical outcomes. These findings suggest that optimization of VB dose–volume parameters may represent a promising marrow-sparing strategy to mitigate treatment-related HT and preserve systemic immune competence during combined chemoradiotherapy and immunotherapy. Further prospective studies are warranted to validate these dosimetric constraints and to clarify their potential role in guiding treatment planning in the immunotherapy era.

## Data Availability

The original contributions presented in the study are included in the article/**Supplementary Material**. Further inquiries can be directed to the corresponding authors.
