# Supplementary material for: Vertebral body dosimetry associated with hematologic toxicities and survival in esophageal squamous-cell carcinoma treated with chemoradiotherapy plus immunotherapy
Source: Front Immunol. 2026 Jul 31;17:1832306. doi: 10.3389/fimmu.2026.1832306 (PMC13473295; doi:10.3389/fimmu.2026.1832306)
Supplement: Supplementary file 1 [file DataSheet1.docx]

**Supplementary Figure S1：Forest plots of VB dose–volume parameters associated with individual hematologic toxicity subtypes.** EQD_2_-based analyses illustrate the relationships between VB dosimetric parameters and grade ≥3 (**A**) leukopenia, (**B**) neutropenia, (**C**) thrombocytopenia, and (**D**) anemia. Corresponding BED-based analyses are shown for grade ≥3 (**E**) leukopenia, (**F**) neutropenia, (**G**) thrombocytopenia, and (**H**) anemia.

*P* values were adjusted for multiple testing using the Benjamini–Hochberg false discovery rate (FDR) procedure. Adj. *P* indicates FDR-adjusted *P* values.
