# Supplementary material for: Vertebral body dosimetry associated with hematologic toxicities and survival in esophageal squamous-cell carcinoma treated with chemoradiotherapy plus immunotherapy
Source: Front Immunol. 2026 Jul 31;17:1832306. doi: 10.3389/fimmu.2026.1832306 (PMC13473295; doi:10.3389/fimmu.2026.1832306)
Supplement: Supplementary file 2 [file DataSheet2.docx]

**Supplementary Figure S2: ROC analyses of VB low-dose parameters for grade ≥3 lymphopenia.** ROC curves illustrating the discriminative performance of (A) V5_EQD₂_, (B) V10_EQD₂_, and (C) V20_EQD₂_ for grade ≥3 lymphopenia.
