# Supplementary material for: Vertebral body dosimetry associated with hematologic toxicities and survival in esophageal squamous-cell carcinoma treated with chemoradiotherapy plus immunotherapy
Source: Front Immunol. 2026 Jul 31;17:1832306. doi: 10.3389/fimmu.2026.1832306 (PMC13473295; doi:10.3389/fimmu.2026.1832306)
Supplement: Supplementary file 3 [file DataSheet3.docx]

**Supplementary Figure S3: Correlation between VB BED dose–volume parameters and post-radiotherapy ALC.** Spearman correlation analyses depict the relationships between (**A**) V5_BED_, (**B**) V10_BED_, and (**C**) V20_BED_ and the post-radiotherapy ALC.
