# Supplementary material for: Vertebral body dosimetry associated with hematologic toxicities and survival in esophageal squamous-cell carcinoma treated with chemoradiotherapy plus immunotherapy
Source: Front Immunol. 2026 Jul 31;17:1832306. doi: 10.3389/fimmu.2026.1832306 (PMC13473295; doi:10.3389/fimmu.2026.1832306)
Supplement: Supplementary file 4 [file SupplementaryFile1.zip › Table S1.DOCX]

**Supplementary Table S1. Acute hematologic toxicities of patients with ESCC.**

| **Variables** | **N** |
| --- | --- |
| **WBC** |  |
| Nadir (10^3^/μL) | 2.24 (1.76–2.74) |
| Grade 0–2 | 95 (60.5%) |
| Grade 3–4 | 62 (39.5%) |
| **ANC** |  |
| Nadir (10^3^/μL) | 1.12 (0.77–1.69) |
| Grade 0–2 | 99 (63.1%) |
| Grade 3–4 | 58 (36.9%) |
| **Platelets** |  |
| Nadir (10^3^/μL) | 113 (82–139) |
| Grade 0–2 | 149 (94.9%) |
| Grade 3–4 | 8 (5.1%) |
| **Hemoglobin** |  |
| Nadir (10^3^/μL) | 10.70 (9.45–11.60) |
| Grade 0–2 | 154 (98.1%) |
| Grade 3–4 | 3 (1.9%) |
| **ALC** |  |
| Nadir (10^3^/μL) | 0.32 (0.24–0.44) |
| Grade 0–2 | 26 (16.6%) |
| Grade 3–4 | 131 (83.4%) |
