# Supplementary material for: Vertebral body dosimetry associated with hematologic toxicities and survival in esophageal squamous-cell carcinoma treated with chemoradiotherapy plus immunotherapy
Source: Front Immunol. 2026 Jul 31;17:1832306. doi: 10.3389/fimmu.2026.1832306 (PMC13473295; doi:10.3389/fimmu.2026.1832306)
Supplement: Supplementary file 4 [file SupplementaryFile1.zip › Table S2.DOCX]

**Supplementary Table S2. The relationship between clinical characteristics and HT3+.**

| **Variables** | **HT3+, N =141 (%)** | **No HT3+, N = 16 (%)** | ***P* value** |
| --- | --- | --- | --- |
| **Age (y)** |  |  | 0.414 |
| <65 | 82 (88.2%) | 11 (11.8%) |  |
| ≥65 | 59 (92.2%) | 5 (7.8%) |  |
| **Gender** |  |  | 1.000 |
| Male | 125 (89.9%) | 14 (10.1%) |  |
| Female | 16 (88.9%) | 2 (11.1%) |  |
| **BMI** |  |  | 0.642 |
| <25 | 102 (88.7%) | 13 (11.3%) |  |
| ≥25 | 39 (92.9%) | 3 (7.1%) |  |
| **ECOG** |  |  | 0.617 |
| 0 | 71 (91.0%) | 7 (9.0%) |  |
| 1 | 70 (88.6%) | 9 (11.4%) |  |
| **Smoking history** |  |  | 0.477 |
| No | 57 (91.9%) | 5 (8.1%) |  |
| Yes | 84 (88.4%) | 11 (11.6%) |  |
| **Drinking history** |  |  | 0.884 |
| No | 59 (89.4%) | 7 (10.6%) |  |
| Yes | 82 (90.1%) | 9 (9.9%) |  |
| **Tumor site** |  |  | 0.077 |
| Cervical | 13 (76.5%) | 4 (23.5%) |  |
| Thoracic | 116 (92.8%) | 9 (7.2%) |  |
| Both | 12 (80%) | 3 (30%) |  |
| **Baseline WBC (10^3^/μL) ^#^** |  |  | 0.979 |
| <6.0 | 71 (89.9%) | 8 (10.1%) |  |
| ≥6.0 | 70 (89.7%) | 8 (10.3%) |  |
| **Baseline ANC (10^3^/μL) ^#^** |  |  | 0.936 |
| <3.7 | 72 (90.0%) | 8 (10.0%) |  |
| ≥3.7 | 69 (89.6%) | 8 (10.4%) |  |
| **Baseline Platelets (10^3^/μL) ^#^** |  |  | 0.579 |
| <212 | 69 (88.5%) | 9 (11.5%) |  |
| ≥212 | 72 (91.1%) | 7 (8.9%) |  |
| **Baseline Hemoglobin (g/μL) ^#^** |  |  | 0.893 |
| <13.0 | 68 (89.5%) | 8 (10.5%) |  |
| ≥13.0 | 73 (90.1%) | 8 (9.9%) |  |
| **Baseline ALC (103/μL) ^#^** |  |  | 0.163 |
| <1.6 | 70 (93.3%) | 5 (6.7%) |  |
| ≥1.6 | 71 (86.6%) | 11 (13.4%) |  |
| **Immunotherapy type** |  |  | 1.000 |
| Anti-PD-1 immune checkpoint | 126 (90.0%) | 14 (10.0%) |  |
| Anti-PD-L1 immune checkpoint | 15 (88.2%) | 2 (11.8%) |  |
| **Immunotherapy timing** |  |  | 0.414 |
| Before, during, and after RT | 46 (95.8%) | 2 (4.2%) |  |
| Before and after RT | 18 (90.0%) | 2 (10.0%) |  |
| Before RT alone | 15 (88.2%) | 2 (11.8%) |  |
| After RT alone | 26 (89.7%) | 3 (10.3%) |  |
| Concurrent RT | 36 (83.7%) | 7 (16.3%) |  |
| **Chemotherapy regimen** |  |  | 0.433 |
| Paclitaxel + Platinum | 14 (82.4%) | 3 (17.6%) |  |
| Nab-paclitaxel + Platinum | 114 (91.9%) | 10 (8.1%) |  |
| Docetaxel + Platinum | 6 (85.7%) | 1 (14.3%) |  |
| Others | 7 (77.8%) | 2 (22.2%) |  |
| **Prescription dose**  **(converted into EQD_2_, Gy)** |  |  | 0.249 |
| <59.2 | 110 (88.0%) | 15 (12.0%) |  |
| ≥59.2 | 31 (96.9%) | 1 (3.1%) |  |

^#^Cutoffs for grouping patients by the median count.
