# Supplementary material for: Vertebral body dosimetry associated with hematologic toxicities and survival in esophageal squamous-cell carcinoma treated with chemoradiotherapy plus immunotherapy
Source: Front Immunol. 2026 Jul 31;17:1832306. doi: 10.3389/fimmu.2026.1832306 (PMC13473295; doi:10.3389/fimmu.2026.1832306)
Supplement: Supplementary file 4 [file SupplementaryFile1.zip › Table S3.DOCX]

**Supplementary Table S3. Association of tumor extent and PTV length with VB dosimetric parameters.**

| **Variable** | **V5_EQD₂_** | **V10_EQD₂_** | **V20_EQD₂_** |
| --- | --- | --- | --- |
| **Tumor length**  (≤5 cm vs >5 cm) | *P* = 0.010 | *P* = 0.008 | *P* = 0.005 |
| **N stage**  (N0–1 vs N2–3) | *P* < 0.001 | *P* < 0.001 | *P* = 0.001 |
| **PTV length** | *P* < 0.001 | *P* < 0.001 | *P* < 0.001 |
