# Supplementary material for: Vertebral body dosimetry associated with hematologic toxicities and survival in esophageal squamous-cell carcinoma treated with chemoradiotherapy plus immunotherapy
Source: Front Immunol. 2026 Jul 31;17:1832306. doi: 10.3389/fimmu.2026.1832306 (PMC13473295; doi:10.3389/fimmu.2026.1832306)
Supplement: Supplementary file 4 [file SupplementaryFile1.zip › Table S4.DOCX]

**Supplementary Table S4. Univariable and multivariable logistic regression analyses of factors associated with immunotherapy response.**

| **Variables** | **Univariate analysis** | | **Multivariate analysis** | |
| --- | --- | --- | --- | --- |
|  | **OR (95% CI)** | ***P* value** | **OR (95% CI)** | ***P* value** |
| **Age** | 1.013 (0.973–1.055) | 0.530 |  |  |
| **Gender** |  |  |  |  |
| Male vs Female | 4.950 (1.738–14.096) | 0.003 | 4.482 (1.260–15.941) | 0.020^†^ |
| **BMI** | 0.939 (0.855–1.032) | 0.190 |  |  |
| **ECOG** |  |  |  |  |
| 1 vs 0 | 1.283 (0.659–2.499) | 0.463 |  |  |
| **Smoking history** |  |  |  |  |
| Yes vs No | 2.436 (1.232–4.816) | 0.010 | 1.700 (0.755–3.828) | 0.200 |
| **Drinking history** |  |  |  |  |
| Yes vs No | 1.445 (0.739–2.826) | 0.282 |  |  |
| **Baseline WBC** | 1.024 (0.890–1.179) | 0.736 |  |  |
| **Baseline ANC** | 1.035 (0.876–1.224) | 0.684 |  |  |
| **Baseline Platelets** | 1.000 (0.995–1.004) | 0.828 |  |  |
| **Baseline Hemoglobin** | 1.087 (0.858–1.377) | 0.488 |  |  |
| **Baseline ALC** | 0.652 (0.328–1.292) | 0.220 |  |  |
| **Immunotherapy type** |  |  |  |  |
| Anti-PD-L1 vs anti-PD-1 | 1.213 (0.403–3.646) | 0.731 |  |  |
| **T stage** |  |  |  |  |
| T1 | Reference |  |  |  |
| T2 | 0.750 (0.055–10.233) | 0.829 |  |  |
| T3 | 1.026 (0.090–11.659) | 0.984 |  |  |
| T4 | 1.167 (0.088–15.458) | 0.907 |  |  |
| **N stage** |  |  |  |  |
| N2-3 vs N0-1 | 1.755 (0.897–3.435) | 0.101 |  |  |
| **Stage group** |  |  |  |  |
| IV vs III | 2.400 (1.214–4.745) | 0.012 | 2.116 (1.016–4.410) | 0.045^†^ |
| **Tumor length** |  |  |  |  |
| >5cm vs ≤5cm | 1.387 (0.711–2.705) | 0.337 |  |  |
| **Chemotherapy regimen** |  |  |  |  |
| Nab-paclitaxel ±  Platinum | Reference |  |  |  |
| Paclitaxel ± Platinum | 0.810 (0.279–2.351) | 0.698 |  |  |
| Docetaxel ± Platinum | 0.589 (0.126–2.762) | 0.502 |  |  |
| Others | 0.353 (0.090–1.390) | 0.137 |  |  |
| **Radiation dose** | 0.990 (0.948–1.034) | 0.657 |  |  |
| **PTV length** | 1.119 (1.043–1.200) | 0.002 | 1.112 (1.033–1.196) | 0.005^†^ |
| **VB dose–volume parameter** |  |  |  |  |
| V5_EQD₂_ | 1.027 (1.009–1.044) | 0.003 | 1.029 (1.010–1.048) | 0.002^†^ |
| V10_EQD₂_ | 1.029 (1.010–1.047) | 0.002 | 1.031 (1.012–1.052) | 0.002^†^ |
| V20_EQD₂_ | 1.030 (1.011–1.050) | 0.002 | 1.033 (1.012–1.054) | 0.002^†^ |

^†^*P* value is significant.
