# Supplementary material for: Vertebral body dosimetry associated with hematologic toxicities and survival in esophageal squamous-cell carcinoma treated with chemoradiotherapy plus immunotherapy
Source: Front Immunol. 2026 Jul 31;17:1832306. doi: 10.3389/fimmu.2026.1832306 (PMC13473295; doi:10.3389/fimmu.2026.1832306)
Supplement: Supplementary file 4 [file SupplementaryFile1.zip › Table S5.DOCX]

**Supplementary Table S5. Univariable and multivariable Cox regression analyses of factors associated with PFS.**

| **Variables** | **Univariate** | | **Multivariable** | |
| --- | --- | --- | --- | --- |
|  | **OR (95% CI)** | **P value** | **OR (95% CI)** | **P value** |
| **Age** | 0.988 (0.961–1.016) | 0.410 |  |  |
| **Gender** |  |  |  |  |
| Male vs Female | 4.396 (1.361–4.195) | 0.013 | 4.113 (1.258–13.448) | 0.019^†^ |
| **BMI** | 0.938 (0.876–0.005) | 0.067 | 0.935 (0.871–1.003) | 0.061 |
| **ECOG** |  |  |  |  |
| 1 vs 0 | 0.966 (0.602–1.551) | 0.887 |  |  |
| **Smoking history** |  |  |  |  |
| Yes vs No | 1.258 (0.772–2.051) | 0.357 |  |  |
| **Drinking history** |  |  |  |  |
| Yes vs No | 1.310 (0.799–2.148) | 0.284 |  |  |
| **Baseline WBC** | 0.987 (0.913–1.066) | 0.732 |  |  |
| **Baseline ANC** | 0.977 (0.883–1.080) | 0.646 |  |  |
| **Baseline Platelets** | 1.001 (0.998–1.004) | 0.613 |  |  |
| **Baseline Hemoglobin** | 0.892 (0.750–1.062) | 0.198 |  |  |
| **Baseline ALC** | 1.055 (0.637–1.750) | 0.834 |  |  |
| **T stage** |  |  |  |  |
| T1 | Reference |  |  |  |
| T2 | 0.298 (0.050–1.795) | 0.186 |  |  |
| T3 | 0.508 (0.123–2.094) | 0.349 |  |  |
| T4 | 0.681 (0.152–3.054) | 0.616 |  |  |
| **N stage** |  |  |  |  |
| N2-3 vs N0-1 | 1.561 (0.951–2.561) | 0.078 | 1.311 (0.771–2.230) | 0.318 |
| **Stage group** |  |  |  |  |
| IV vs III | 2.132 (1.236–3.676) | <0.001 | 2.132 (1.236–3.676) | 0.006^†^ |
| **Tumor length** |  |  |  |  |
| >5cm vs ≤5cm | 1.273 (0.791–2.050) | 0.320 |  |  |
| **Chemotherapy regimen** |  |  |  |  |
| Nab-paclitaxel ±  Platinum | Reference |  |  |  |
| Paclitaxel ± Platinum | 0.557 (0.239–1.300) | 0.176 |  |  |
| Docetaxel ± Platinum | 1.078 (0.261–4.454) | 0.917 |  |  |
| Others | 1.103 (0.343–3.542) | 0.869 |  |  |
| **Radiation dose** | 0.943 (0.945–1.002) | 0.070 | 0.969 (0.939–1.001) | 0.054 |
| **PTV length** | 1.060 (1.014–1.107) | 0.010 | 1.040 (0.993–1.088) | 0.097 |
| **VB dose–volume parameter** |  |  |  |  |
| V5_EQD₂_ | 1.013 (1.002–1.025) | 0.023 | 1.008 (0.997–1.021) | 0.166 |
| V10_EQD₂_ | 1.015 (1.002–1.027) | 0.018 | 1.010 (0.997–1.022) | 0.137 |
| V20_EQD₂_ | 1.014 (1.001–1.027) | 0.034 | 1.009 (0.996–1.022) | 0.176 |

^†^*P* value is significant.
