# Supplementary material for: Vertebral body dosimetry associated with hematologic toxicities and survival in esophageal squamous-cell carcinoma treated with chemoradiotherapy plus immunotherapy
Source: Front Immunol. 2026 Jul 31;17:1832306. doi: 10.3389/fimmu.2026.1832306 (PMC13473295; doi:10.3389/fimmu.2026.1832306)
Supplement: Supplementary file 4 [file SupplementaryFile1.zip › Table S6.DOCX]

**Supplementary Table S6. Univariable and multivariable Cox regression analyses of factors associated with OS.**

| **Variables** | **Univariate** | | **Multivariable** | |
| --- | --- | --- | --- | --- |
|  | **OR (95% CI)** | **P value** | **OR (95% CI)** | **P value** |
| **Age** | 0.997 (0.960–1.034) | 0.860 |  |  |
| **Gender** |  |  |  |  |
| Male vs Female | 6.919 (0.946–50.614) | 0.057 | 4.352 (0.556–34.043) | 0.161 |
| **BMI** | 0.899 (0.821–0.984) | 0.021 | 0.923 (0.834–1.020) | 0.117 |
| **ECOG** |  |  |  |  |
| 1 vs 0 | 0.988 (0.543–1.798) | 0.968 |  |  |
| **Smoking history** |  |  |  |  |
| Yes vs No | 1.810 (0.954–3.436) | 0.069 | 0.932 (0.378–2.296) | 0.879 |
| **Drinking history** |  |  |  |  |
| Yes vs No | 2.110 (1.094–4.071) | 0.026 | 1.920 (0.757–4.873) | 0.170 |
| **Baseline WBC** | 0.968 (0.868–1.080) | 0.561 |  |  |
| **Baseline ANC** | 0.972 (0.852–1.108) | 0.670 |  |  |
| **Baseline Platelets** | 1.003 (1.000–1.007) | 0.052 | 1.003 (0.999–1.006) | 0.135 |
| **Baseline Hemoglobin** | 0.918 (0.737–1.145) | 0.449 |  |  |
| **Baseline ALC** | 0.869 (0.464–1.628) | 0.661 |  |  |
| **T stage** |  |  |  |  |
| T1 | Reference |  |  |  |
| T2 | 0.273 (0.038–1.971) | 0.198 |  |  |
| T3 | 0.338 (0.079–1.440) | 0.142 |  |  |
| T4 | 0.436 (0.091–2.100) | 0.301 |  |  |
| **N stage** |  |  |  |  |
| N2-3 vs N0-1 | 1.540 (0.827–2.866) | 0.174 |  |  |
| **Stage group** |  |  |  |  |
| IV vs III | 2.192 (1.168–4.114) | 0.015 | 1.671 (0.873–3.198) | 0.121 |
| **Tumor length** |  |  |  |  |
| >5cm vs ≤5cm | 0.916 (0.502–1.671) | 0.775 |  |  |
| **Chemotherapy regimen** |  |  |  |  |
| Nab-paclitaxel ±  Platinum | Reference |  |  |  |
| Paclitaxel ± Platinum | 0.907 (0.378–2.174) | 0.826 |  |  |
| Docetaxel ± Platinum | 1.645 (0.221–12.235) | 0.627 |  |  |
| Others | 1.558 (0.371–6.544) | 0.545 |  |  |
| **Radiation dose** | 1.526 (0.644–3.617) | 0.337 |  |  |
| **PTV length** | 1.085 (1.027–1.146) | 0.003 | 1.074 (1.013–1.139) | 0.017^†^ |
| **VB dose–volume parameter** |  |  |  |  |
| V5_EQD₂_ | 1.023 (1.008–1.038) | 0.003 | 1.020 (1.004–1.037) | 0.013^†^ |
| V10_EQD₂_ | 1.024 (1.008–1.040) | 0.003 | 1.021 (1.004–1.039) | 0.015^†^ |
| V20_EQD₂_ | 1.023 (1.006–1.041) | 0.007 | 1.019 (1.002–1.037) | 0.033^†^ |

^†^*P* value is significant.
